# Mapping Non-Coding RNAs in Space and Time: Another Weapon to Dissect Intra-Tumor Heterogeneity in Cancer Progression

**DOI:** 10.3390/cancers15123181

**Published:** 2023-06-14

**Authors:** Mario Cioce, Andrea Marra, Daniela Rutigliano, Vito Michele Fazio

**Affiliations:** 1 Laboratory of Molecular Medicine and Biotechnology, Department of Medicine, University Campus Bio-Medico of Rome, 00128 Rome, Italy; m.cioce@unicampus.it (M.C.); andrea.marra@unicampus.it (A.M.); d.rutigliano@unicampus.it (D.R.); 2 Institute of Translational Pharmacology, National Research Council of Italy (CNR), 00133 Rome, Italy

It is increasingly clear that Intratumor heterogeneity (ITH) fuels tumor evolution, matching the concept of cancer as a heterogeneous ecosystem of spatially and temporally modulated cell subpopulations, which exploits dynamic strategies to hijack local and systemic resources and tissue(s) space [1]. ITH has a direct influence on the mechanisms of tumor immune escape, and it is tightly correlated with tumor metastatic propensity and acquired therapeutic resistance [2,3]. At a molecular level, the non-coding genome, and specifically lncRNAs may play a pivotal role as determinants of ITH [4]. LncRNAs account for about 70% of the human genome and are untranslated, polyadenylated transcripts of >200 nt. The lncRNAs may control gene expression at both transcriptionally and post-transcriptionally levels, in a subcellular localization-dependent manner. In the nucleus, lncRNAs affect chromatin structure at least by tethering epigenetic modifiers on the DNA. In the cytoplasm, cytoplasmic lncRNAs-mediated gene regulation occurs by competing with specific microRNAs for binding to endogenous RNAs, thereby affecting translation [5]. An example of such a dual functioning is provided by the lncRNA CCAT1-L, which acts as an oncogene by binding to CCCTC-binding transcription factor (CTCF), thus affecting chromatin bound c-MYC oncoprotein. Conversely, cytoplasmic CCAT1 sponges several tumor suppressive miRNAs, allowing miRNA-targeted mRNAs to feed mechanisms of tumor proliferation, invasion and metastasis [6].

Distinct lncRNA/miRNA/protein regulatory axes have been described in many tumor subtypes, including colorectal cancer (CRC) [7]. As well, reported by Marie Rajtmaierova and colleagues in a recent issue of Cancers [8], these molecular switches can regulate multiple aspects of tumor biology, such as the tumor microenvironment/TME composition or the resistance to chemotherapy. In this regard, it has been shown that lncRNA SCARNA2, found to be upregulated in CRC tissues, can directly activate the miR-342-3p-EGFR/BCL2 axis for escaping 5-fluorouracil cytotoxicity [9]. Other evidence have demonstrated that such regulatory axes may indirectly support tumor growth through the downstream recruitment of master transcriptional regulators, such as c-MYC [10] or mutant p53 [11].

At a microenvironmental level, lncRNA/miRNA axes may affect immunity, as suggested by recent works which identify clinically relevant lncRNA immune-related signatures in tumors. According to this, it has been proposed that a lncRNA signature of tumor immune heterogeneity can predict the risk of distant metastasis in loco-regionally advanced nasopharyngeal carcinomas [12], or the overall survival and immunotherapy response in CRC [13].

At an epigenetic level, it should be noted that an intimate relationship between lncRNA and nuclear domains can be confidently traced, with lncRNAs mediating nuclear phase separation thereby affecting spatial gene expression during tissue development [7]. This is a crucial aspect in biology, since the differentiation state of eukaryotic cells is both an effect and a determinant of cell-type specific chromatin structure.

Based on these evidence, future research efforts should aim to correlate lncRNA expression and ITH, by conducting a spatiotemporal analysis of lncRNA/miRNAs dynamics within cell subpopulations in the tumor, at a multilevel resolution. According to this, it will be of interest to gain insight on how lncRNAs hubs may fine tune cell states and phenotypes under adaptive stress responses (such as chemo- and radiotherapy).

Importantly, we have described specific waves of microRNA clusters from EphA2- and EphB2-expressing CRC human and murine tumors, which have allowed us to define some stemness-related and progression-related functions to EphB2 and EphA2, respectively [14]. The use of flow-cytometry-assisted cell sorting of tumor subpopulations coupled to the microRNA analysis has allowed us to obtain initial hints of the ITH in such a system. The analysis of lncRNA networks and of their functional modulation of miRNA distribution in the same experimental settings may be of relevance, as it has been shown in the past in other cancer histologies, such as mesothelioma [15].

A key question to pose herein is what mediates the cancer cells–TME crosstalk by cargoing lnc- and mi-RNAs. An obvious and important candidate for such a function is represented by the exosomes [16,17]. For example, in another recent issue of Cancers, Xulin Zhou and colleagues have shown that exosomes derived from pancreatic cancer cells promoted lymphangiogenesis in vitro and in vivo, and that this resulted from the downregulation of the lncRNA ABHD11-AS1 in the lymphatic endothelial cells (LECs). Indeed, at a steady state, ABHD11-AS1 enhanced the proliferation, migration, and ability of the LECs to form tubes [18].

Another foreseeable area of investigation is about how to exploit such regulatory circuits for therapeutic purposes. The pleiotropic nature and mode of action of lncRNAs may somewhat discourage precision-based approaches. However, this apparent downside may turn out to be useful if one considers using natural compounds, for which the mechanism of action is broad by definition. For example, this is the case of celastrol and butein—both naturally occurring compounds capable of modulating a large fraction of ncRNAs [15,19].

Finally, due consideration must be devoted to the models. Are we using clinically relevant models to study and dissect lncRNA functions? To date, the vast majority of the published research employs a two-dimensional culturing of the cells. Albeit useful, this may limit the clinical relevance of the gained knowledge., since many modulators of lncRNA behavior, including matrix stiffness [20], presence of the tumor-microenvironment (TME) cell populations, and growth of endothelial structures may not be easily reproduced in conventional cultures. Organoids are 3D, self-organized structures which are accurate representations of both structural and functional characteristics of adult tissues. Organoids are generally devoid of TME components or immune infiltrates. Ideally, an effort of co-culturing the organoids with both TME components and peripheral blood mononuclear cells could represent an advancement to investigate lncRNA dynamics in light of the tumor cell clone–microenvironment interactions. Efforts in such a direction are ongoing in our and other labs.
